# Synthesis and application of magnetic@layered double hydroxide as an anti-inflammatory drugs nanocarrier

**DOI:** 10.1186/s12951-020-00718-y

**Published:** 2020-10-29

**Authors:** Vahid Yousefi, Vahideh Tarhriz, Shirin Eyvazi, Azita Dilmaghani

**Affiliations:** 1 Molecular Medicine Research Center, Biomedicine Institute, Tabriz University of Medical Sciences, Tabriz, Iran; 2 Department of Biotechnology, School of Advanced Technologies in Medicine, Shahid Beheshti University of Medical Sciences, Tehran, Iran; 3 Drug Applied Research Center, Tabriz University of Medical Sciences, Tabriz, Iran; 4 Department of Pharmaceutical Biotechnology, Faculty of Pharmacy, Tabriz University of Medical Sciences, Tabriz, Iran

**Keywords:** Layered double hydroxide, Nanostructure, Iron oxide nanoparticle, Anti-inflammatory drugs, Drug delivery

## Abstract

**Background:**

Magnetic nanocomposites with a core–shell nanostructure have huge applications in different sciences especially in the release of the drugs, because of their exclusive physical and chemical properties. In this research, magnetic@layered double hydroxide multicore@shell nanostructure was synthesized by the facile experiment and is used as novel drug nanocarrier.

**Methods:**

Magnetic nanospheres were synthesized by a facile one-step solvothermal route, and then, layered double hydroxide nanoflakes were prepared on the magnetic nanospheres by coprecipitation experiment. The synthesized nanostructures were characterized by FTIR, XRD, SEM, VSM, and TEM, respectively. After intercalation with Ibuprofen and Diclofenac as anti-inflammatory drugs and using exchange anion experiment, the basal spacing of synthesized layered double hydroxides was compared with brucite nanosheets from 0.48 nm to 2.62 nm and 2.22 nm, respectively.

**Results:**

The results indicated that Ibuprofen and Diclofenac were successfully intercalated into the interlay space of LDHs via bridging bidentate interaction. In addition, in-vitro drug release experiments in pH 7.4, phosphate-buffered saline (PBS) showed constant release profiles with Ibuprofen and Diclofenac as model drugs with different lipophilicity, water solubility, size, and steric effect.

**Conclusion:**

The Fe_3_O_4_@LDH-ibuprofen and Fe_3_O_4_@LDH-diclofenac had the advantage of the strong interaction between the carboxyl groups with higher trivalent cations by bridging bidentate, clarity, and high thermal stability. It is confirmed that Fe_3_O_4_@LDH multicore-shell nanostructure may have potential application for constant drug delivery.
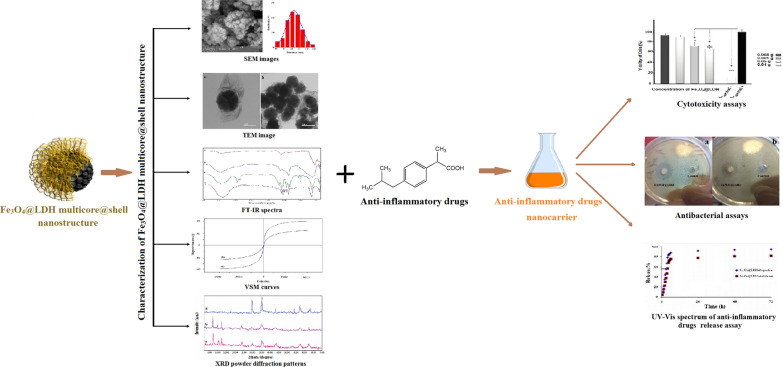

## Background

In recent years, magnetic nanocomposites with a core–shell nanostructure have attracted increasing attention because of their exclusive physical and chemical properties and huge possible applications in different areas such as extraction, the release of drug, medicine, mechanical aspects, etc. [1–5]. Several performance-particulars with high surface shells have been prepared so far on the iron oxide nanospheres, significantly growing the magnetic core@shell nanostructure area [6–8]. Inorganic nanomaterials are commonly used because of their low cost, high surface area, easy availability, and easy preparation. Layered double hydroxides (LDHs), recognized as anionic clays or brucite-like compounds, are two important sub-classes of ionic layered materials. LDHs are represented with the general formula of [M(II)_1−x_M(III)_x_·(OH)_2_]^x+^ [A^n−^]_x/n_·mH_2_O, where M^2+^ and M^3+^ are respectively di- and trivalent metal cations, and A is n-valent interlayer guest anion. The primary constituents of LDHs are the charged layers that provide diverse chemical compounds with versatile usability, for example, biocompatibility, adsorption, intercalation, and ion exchange [9–11]. These are the bases of LDHs diverse technology applications in a variety of fields including medicine, polymer industries, electrochemistry, food, catalysis, drug delivery separation, and more. In comparison to other drug delivery mechanisms, which show low circulation stability, poor bioavailability, and drug degradation, LDH as exquisite drug nano-carriers are comparatively economical with little toxicity for the cells and biocompatibility [12]. Moreover, ease of production and large capacity for affective drug transportation make them ideal nano-carreirs [10]. Even so, layered double hydroxide nanoparticles are easily aggregated in phosphate buffered saline solution that reduces the number of proper sized nanoparticles available for internalization and, as a result, affect the delivery efficiency [13]. To overcome the current disadvantage, designing and application of anti-aggregation materials such as nanostructure that exploit porosity and high surface are an urgent demand, offering limited control over the morphology, surface area, and particles size that powerfully define the practical performances. Additionally, the achievements of previous studies in this scope encouraged us to use LDH nanostructures, as a suitable drug carrier. LDHs also protect the cargo from environmental modifications and degradation. In addition, they enhance the loading capacity, stability, and penetration aptitude of the cargoes [11, 14]. Some studies have indicated that LDHs are simply aggregated in a phosphate-buffered solution (PBS), decreasing the number of properly sized LDHs available for internalization and improving the efficiency of delivery [14]. Likewise, LDHs are able to infiltrate into the cells and stabilize the drugs and biomolecules within the interlayer. Consequently, as excellent preserving molecules, LDHs protect the loaded molecules from damage, degradation, and alternation and also enhance the loading density, chemicophysical stability, and penetration ability of the loaded drug [15–17]. On the other hand, LDHs containing magnesium and aluminum have already been used as an antacid and antipepsin agent; hence, LDH is quite biocompatible.

In order to pass through the current drawbacks, designing and synthesis of anti-aggregation compounds such as nanostructures that utilize porosity and high surface are an instant demand. The nanostructures should propose restricted control over the morphology, pore architectures, surface area, and particles size that strongly explain the practical performances. Importantly, LDHs carry magnesium and aluminum to display antacid and antipepsin function; therefore, LDH is identified as a fully biocompatible combination. Accordingly, in this research, Fe_3_O_4_@LDH multicore@shell nanostructure was synthesized by the facile experiment and used as a novel drug nanocarrier. The carriers were characterized using XRD, FT-IR, TEM, VSM, and SEM in order to display chemical structure and morphology.

## Materials and methods

### Materials and reagents

Iron(III) chloride, ethylene glycol, magnesium nitrate hexahydrate, ammonium acetate, aluminum nitrate nonahydrate, and all solvents were purchased from the Sigma-Aldrich or Merck companies.

### ***Synthesis of uniform-size Fe***_***3***_***O***_***4***_*** nanospheres***

8 mmol Iron(III) chloride was dissolved in ethylene glycol (45 mL), and then, 45 mmol of ammonium acetate was added under rapid stirring. Afterwards, the resulting mixture was solvothermal treated at 190 °C for 8 h. Before collecting the powder via centrifugation, it was washed and dried at 70 °C overnight.

### ***In-situ synthesis of Fe***_***3***_***O***_***4***_***@LDH multicore@shell nanostructure***

The experimental procedure for the preparation of layered double hydroxide nanoflake on Fe_3_O_4_ was similar to that described in the literature. Briefly, 0.3 g of the synthesized Fe_3_O_4_ nanoparticles was spread into 200 ml water and methanol (1:1), ultrasonically agitating for 20 min to achieve a homogeneous suspension. Then, a 200 ml solution made of 2.6 g of sodium carbonate and 3.2 g sodium hydroxide in water and methanol solution (1:1) were added into the solution until pH = 10 was obtained. Formerly, 200 ml water and methanol solution (1:1) containing 2.25 g aluminum nitrate nonahydrate and 2.6 g magnesium nitrate hexahydrate were added to the prepared suspension by controlling and maintaining pH at 10 through the addition of an alkaline solution simultaneously. The semiliquid mixture was aged at 70 °C overnight. The product was isolated using an external magnet, and the precipitate was rinsed three times with water and ethanol and then dried in an oven at 70 °C for 12 h. Finally, to load each drug, 3 g of synthesized-nanostructure was immersed in 100 ml distilled water containing 3 g of the desired drug (Ibuprofen and Diclofenac) and 1 g of potassium hydroxide with pH control at 9 at room temperature for 24 h [18].

### ***Drug release from drug-containing Fe***_***3***_***O***_***4***_***@LDH multicore@shell nanostructure***

The drug-containing Fe_3_O_4_@LDH multicore@shell nanostructures (0.2 g) were blended with 100 ml of PBS at 120 rpm, pH 7.4, and 37 °C. About 5 ml of solution was disposed and immediately changed with an equal volume of fresh PBS at the same time intervals for keeping the volume constant. The uninvolved solution was centrifuged to remove the Fe_3_O_4_@LDH multicore@shell nanostructures and correctly diluted before the measurement of Ibuprofen and Diclofenac released absorbance via UV–vis spectrophotometer at 264 nm and 276 nm, respectively; the amount released was calculated by means of a standard curve (Additional file 1: Figs. S1 and S2).

### Characterization

FT-IR spectra of the materials were written down over the range of 400–4000 cm^−1^ regions using a Bruker Tensor 27 series FT-IR spectrometer. Powder X-ray diffraction patterns of the samples were recorded in the range of 2°–70° on a Siemens D5000 X-ray Diffractometer, using CuKα radiation (λ = 1.5418 Å) at 30 kV and 30 mA. The morphology of the nanomaterial specimens was observed using the SEM (MIRA3-TESCAN) and transmission electron microscope (Philips CM30). The rate of the absorbance of the drugs was measured by UV–vis spectroscopy (UV-1700 Pharma Spec, Shimadzu).

### ***Antibacterial assays of Fe***_***3***_***O***_***4***_***@LDH***

For testing the antimicrobial feature of Fe_3_O_4_@LDH multicore-shell nanostructure, the gram positive bacterium “*Bacillus cereus strain* ATCC11778^T^” and the gram negative bacteria including *Escherichia coli* strain O157^T^ and *Klebsiella pneumonia* strain PTCC10031^T^ were chosen as the human pathogenic microorganisms for antibacterial activity test. About 250 μg/ml of Fe3O4@LDH was added into 6 cm wells in the Mueller Hinton Agar plates. Then, 0.5 McFarland standard (10^8^ cfu/ml) of the bacteria suspensions was prepared by dilution in Mueller Hinton Broth and slightly spread on the plates. The plates were incubated at 37 °C for 24 h. Methanol solvent (100 μg/ml) was used as negative control. Each experiment was performed in triplicate, and the diameter of the inhibition zones was measured and calculated [19].

### ***Cytotoxicity assays of Fe***_***3***_***O***_***4***_***@LDH***

For testing the cytotoxicity of Fe_3_O_4_@LDH on eukaryotic cells, MTT assays were performed to measure cell viability in the presence of the nanoparticles. Immortalized mouse myoblast cells (C2C12 cells) were seeded at a density of 0.5 × 10^4^ cells per well in 200 ml Dulbecco's modified eagle (DMEM) medium + 10% fetal bovine serum (FBS) with two different concentrations (0.001 and 0.005 g) of Fe_3_O_4_@LDH in 96 well plates. After incubation for 48 h, the media were replaced with fresh culture media containing MTT solution (0.5 mg/ml), and the cells were incubated for an additional 4 h at 37 °C. The absorbance was measured at 570 nm using a spectrophotometric microplate reader (Biotek, EL × 800) [20]. The cells were seeded without nanoparticles as a positive control, and DMSO treated cells were tested as a negative control. One-way ANOVA by graphpad prism ± S.E.M. at P-value < 0.05 was used for data analysis [18].

## Results and discussion

The Fe_3_O_4_@LDH multicore-shell nanostructure was synthesized in two steps: firstly, magnetite nanoparticles were synthesized by solvothermal and Ostwald ripening method, and then layered double hydroxide nanoflakes were prepared on the magnetic nanoparticles by in-situ coprecipitation method and used as the new nanocarrier. Ethylene glycol was used for three reasons: solvent, reduction agent of Fe^3+^ to Fe^2+^, and synthesis of nanoparticles with monodisperse nanoparticles [21].

### Characterization of synthesized nanomaterials

**Fig. 1:**
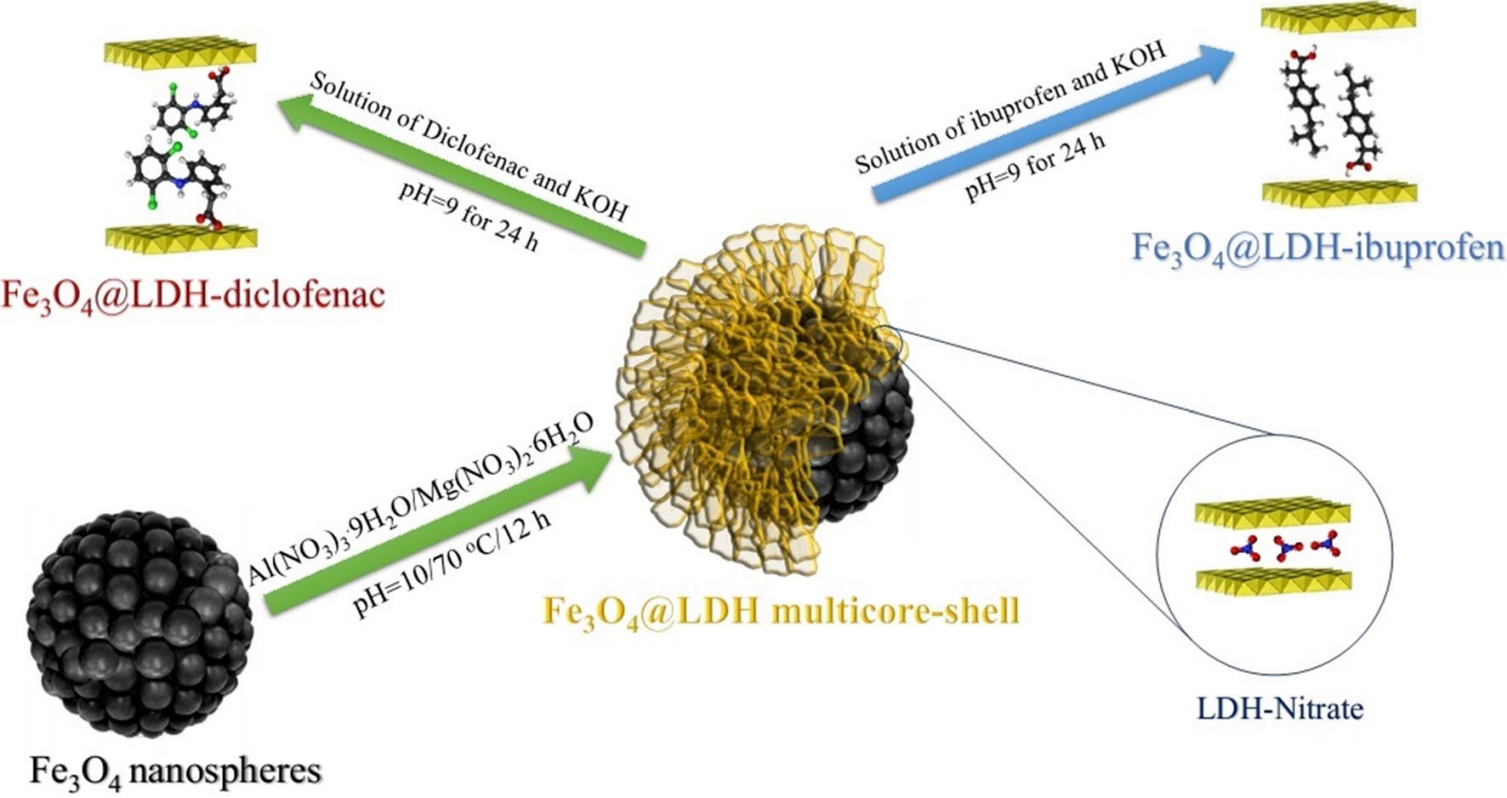
Schematic representation of preparation of Fe_3_O_4_@LDH multicore-shell nanostructure before and after the intercalation of the anti-inflammatory drugs

**Fig. 2:**
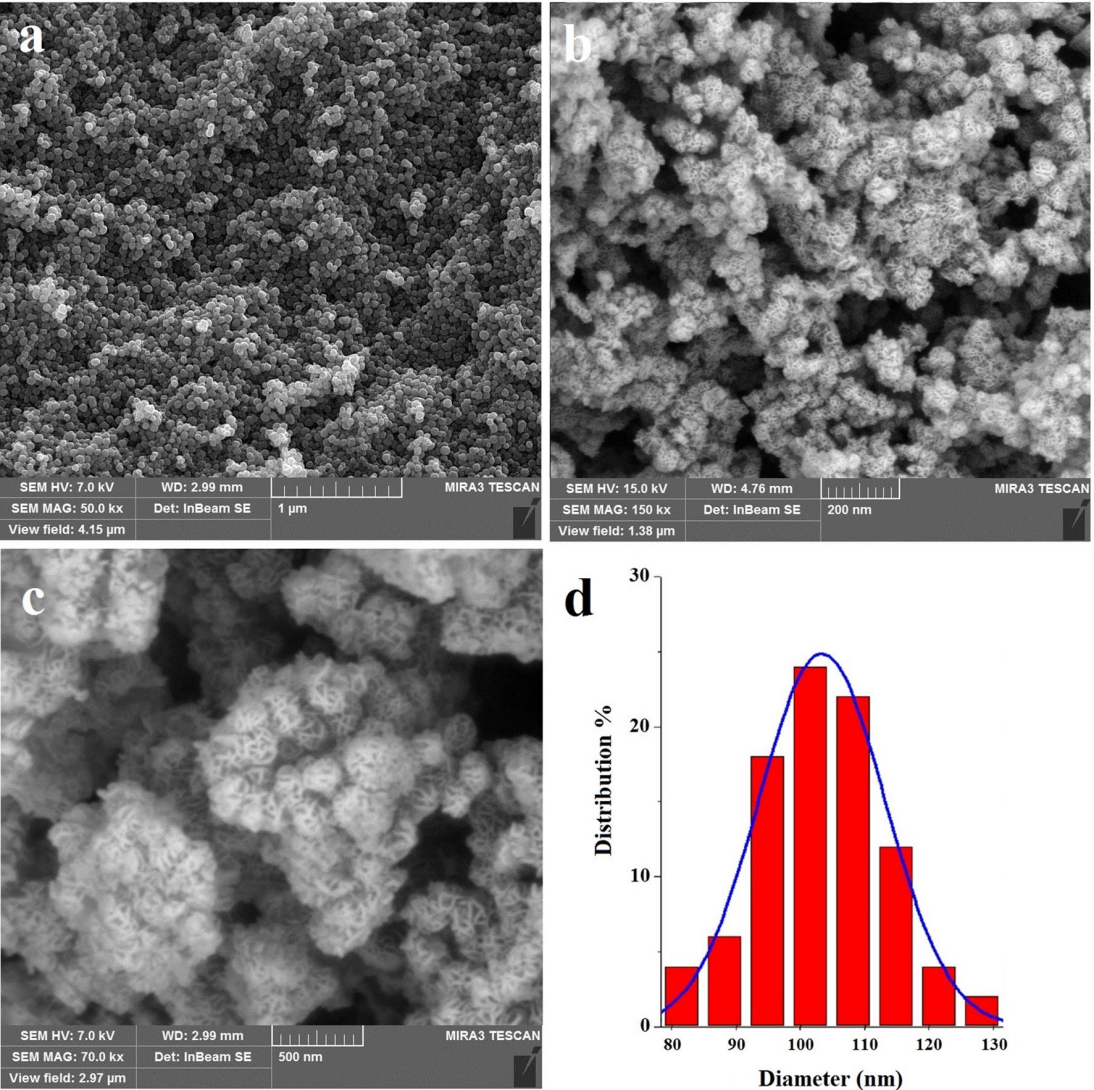
The SEM images of **a** Fe_3_O_4_ nanospheres and **b** Fe_3_O_4_@LDH multicore-shell nanostructure, **c** Fe_3_O_4_@LDH-ibuprofen and **d** the diameter of the Fe_3_O_4_ nanospheres

Figure 1 depicts the schematic of the synthesis of Fe_3_O_4_@LDH multicore@shell nanostructure. Figure 2 confirms and demonstrates surface morphologies and also determines particle sizes of (a) Fe_3_O_4_ nanospheres, (b) Fe_3_O_4_@LDH multicore-shell nanostructure, and (c) Fe_3_O_4_@LDH-ibuprofen. Figure 2a, b obviously display the monodisperse structure of Fe_3_O_4_ nanospheres and Fe_3_O_4_@LDH multicore-shell nanostructure. The diameter of the Fe_3_O_4_ nanostructure is determined 80–130 nm, and the thickness of the LDH nanoflakes shell is about 70–110 nm (Fig. 2d). Furthermore, the Fe_3_O_4_@LDH-ibuprofen, has a morphology, even after loading of drug into the LDH layer structure (Fig. 2c).

**Fig. 3:**
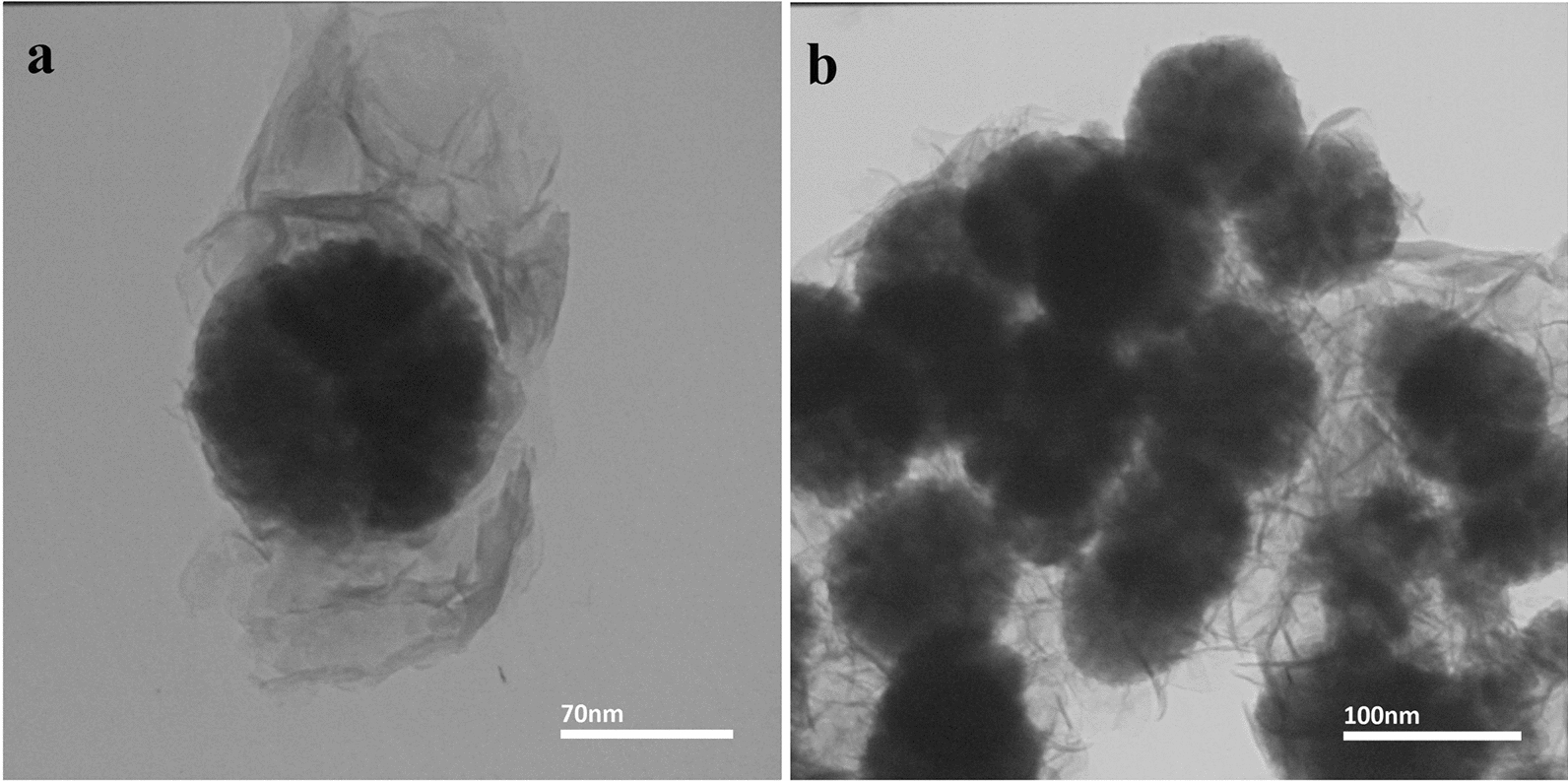
The TEM image of **a** Fe_3_O_4_@LDH multicore-shell nanostructure and **b** Fe_3_O_4_@LDH-ibuprofen, as the drug-loading nanostructure. The multicore, and core–shell structure of nanoparticles is stable completely after loading of the drug

The TEM was used to prove the core–shell structures and to show the porous and multicore nanoparticles created by the Ostwald ripening method. This method leads to formation of magnetic sphere particles in self-assembly form from smaller particles. According to the obtained TEM image, the size of core-forming nanoparticles is 10–13 nm and the size of magnetic spheres is approximately 80–130 nm. With regard to (Fig. 3a) Fe_3_O_4_@LDH multicore-shell nanostructure and (Fig. 3b) Fe_3_O_4_@LDH-ibuprofen, as the drug-loading nanostructure. Figure 3b confirms that the multicore and core–shell structure of nanoparticles are completely stable after loading of the drug.

**Fig. 4:**
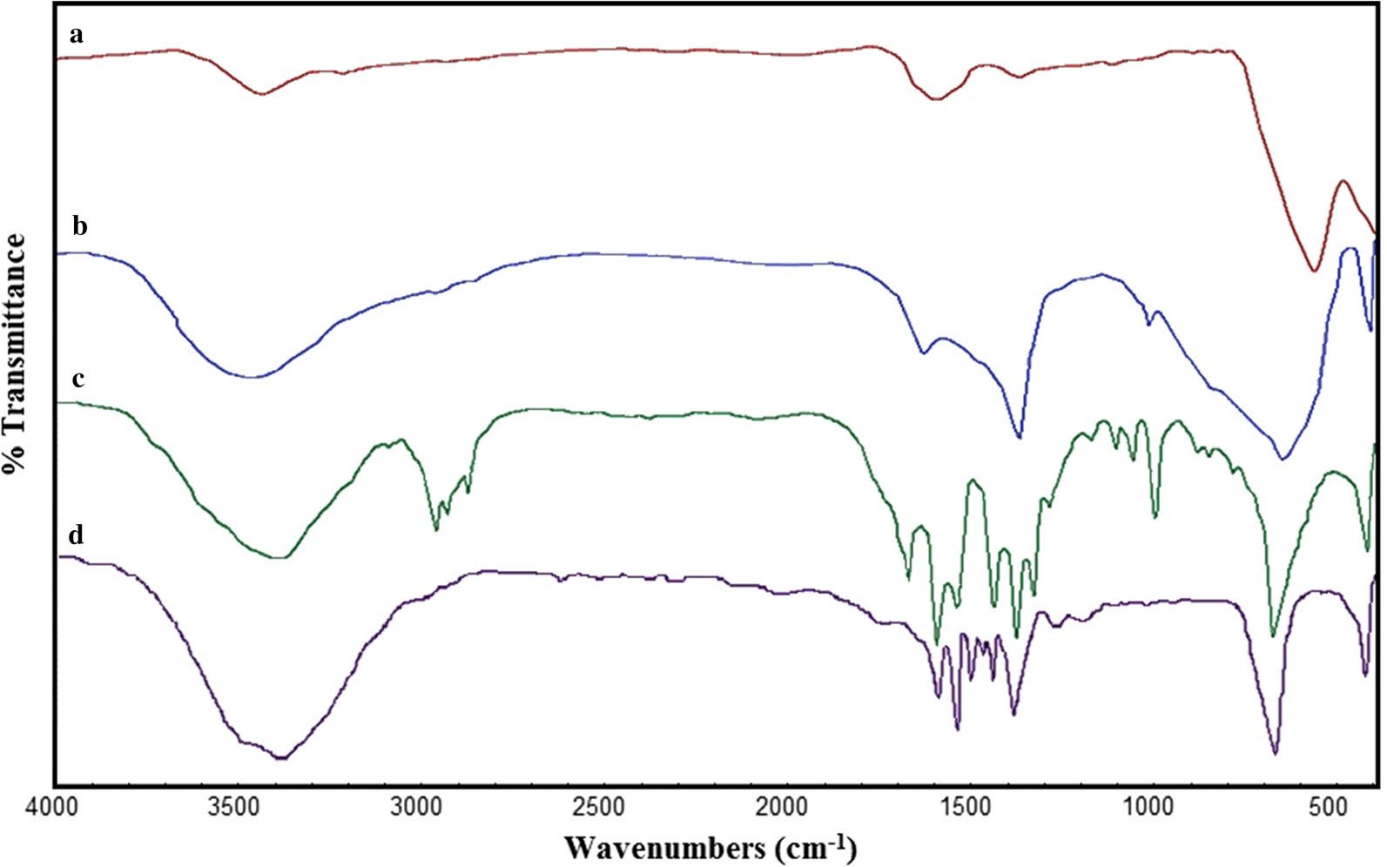
FT-IR spectra of **a** Fe_3_O_4_ nanospheres, **b** Fe_3_O_4_@LDH multicore-shell nanostructure, **c** Fe_3_O_4_@LDH-ibuprofen and **d** Fe_3_O_4_@LDH-diclofenac

The Fourier transform infrared spectra of (a) Fe_3_O_4_ nanospheres, (b) Fe_3_O_4_@LDH multicore-shell nanostructure, (c) Fe_3_O_4_@LDH-ibuprofen, and (d) Fe_3_O_4_@LDH-diclofenac are shown in Fig. 4. The FT-IR spectra of Fe_3_O_4_ nanospheres (Fig. 4a), two highest peaks linked to metal–oxygen bonds, were observed. The first band detected in the range of 385–540 cm^−1^ is normally apportioned to octahedral–metal stretching, whereas the highest one detected in the 500–600 cm^−1^ range is consistent with basic stretching vibrations of the metal at the tetrahedral site. The higher frequency band at 574 cm^−1^ and lower frequency band at 448 cm^−1^are assigned to the tetrahedral and octahedral, respectively. Additionally, the peak at ~ 3360 cm^−1^ is attributed to the stretching vibrations of hydroxyl allocated to hydroxyl absorbed by magnetic nanospheres, and the existence of water is evidenced by the appearance of the bending mode at 1645 cm^−1^ and the stretching mode at 3476 cm^−1^. Compared with the spectrum of Fe_3_O_4_@LDH multicore-shell nanostructure with Fe_3_O_4_@LDH-ibuprofen and Fe_3_O_4_@LDH-diclofenac, there are particular similar peaks in their spectra (Fig. 4c, d). The principal peaks were between 2800 and 3000 cm^−1^ due to the alkyl stretching of drugs, especially in Ibuprofen due to the existence of many methyl groups in its structure compared with Diclofenac. Two peaks also appeared at approximately 1421 and 1576 cm^−1^, recognized to the symmetric and asymmetric stretch of the carboxyl group, respectively. The interaction between the metal atom and the carboxylate groups was classified into three types: monodentate, bridging, and chelating [22, 23]; the major difference (200–320 cm^−1^) was related to the monodentate interaction, and the lowest difference (< 110 cm^−1^) was for the chelating bidentate. The medium-range difference (140–190 cm^−1^) was for the bridging bidentate. The Δ (1576–1421 = 155 cm^−1^) was ascribed as bridging bidentate. Moreover, the peaks at 1440 and 1519 cm^−1^ are related to C–C stretching vibration in benzene rings. These outcomes provided subsequent assistance that Ibuprofen and Diclofenac have been loaded into the layered double hydroxide nanoflakes in the anionic form.

**Fig. 5:**
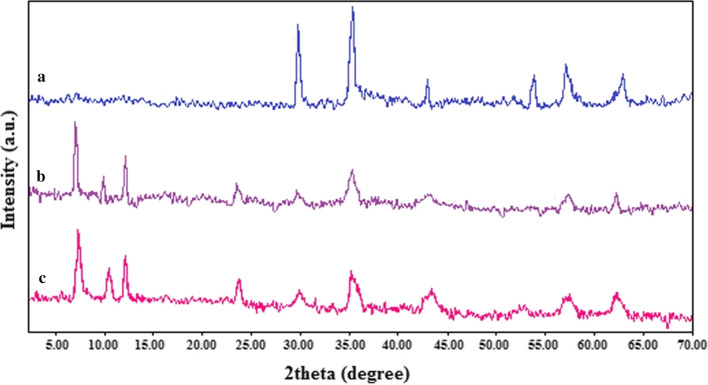
The XRD powder diffraction patterns of **a** Fe_3_O_4_ nanospheres, **b** Fe_3_O_4_@LDH-ibuprofen, **c** Fe_3_O_4_@LDH-diclofenac

The XRD patterns of the Fe_3_O_4_ nanospheres (Fig. 5a) in the 2θ range of 2–70° are shown in Fig. 5. Besides, with loading the drugs, the regenerated matrix indicates representative diffraction peaks of the LDH-drugs, representing two sharp basal reflections indexed as (003) and (006) reflections in line with the well-crystallized lamellar construction in synthesized nanocarrier with 3R rhombic proportion. The important diffraction peaks of Fe_3_O_4_@LDH-ibuprofen (Fig. 5b) and Fe_3_O_4_@LDH-diclofenac (Fig. 5c) are achieved at 2θ value of 7.6° and 7.8°. The d_003_ spacing of Fe_3_O_4_@LDH-ibuprofen and Fe_3_O_4_@LDH-diclofenac were found to be 2.62 nm and 2.22 nm, respectively.

**Fig. 6:**
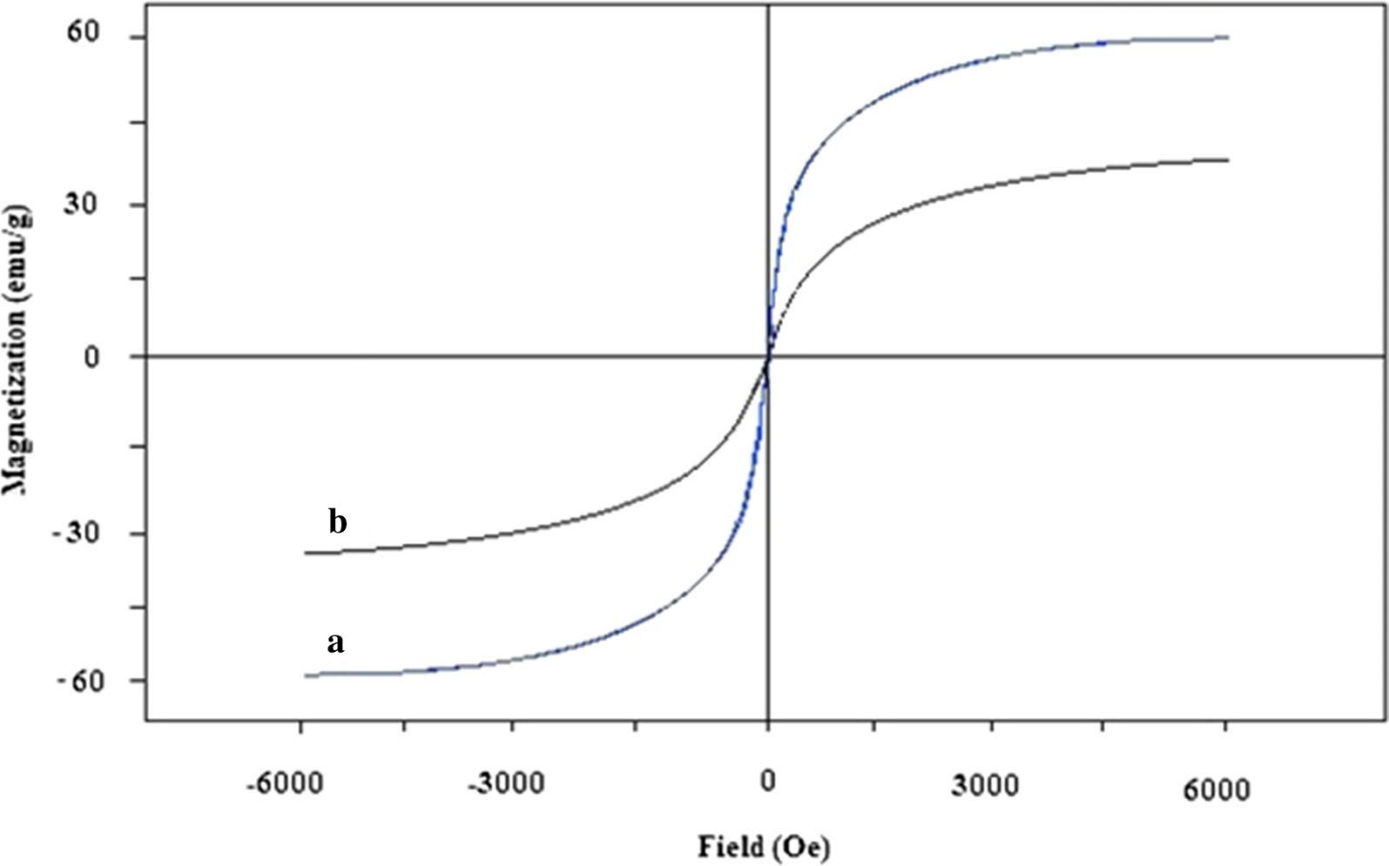
The VSM curves of **a** Fe_3_O_4_ nanospheres and **b** Fe_3_O_4_@LDH multicore-shell nanostructure

The magnetic properties of magnetic nanospheres (Fig. 6a) and Fe_3_O_4_@LDH multicore-shell nanostructure (Fig. 6b) were specified using a vibrating sample magnetometer (VSM). The magnetic saturation values of the magnetic nanospheres and Fe_3_O_4_@LDH multicore-shell nanostructure were 59 and 32 emu/g, respectively. After the LDH shell packing of Fe_3_O_4_ (curve (b)), the saturated magnetization of the Fe_3_O_4_@LDH multicore-shell nanostructure decreases because of the shield of the LDH nanoflakes.

**Fig. 7:**
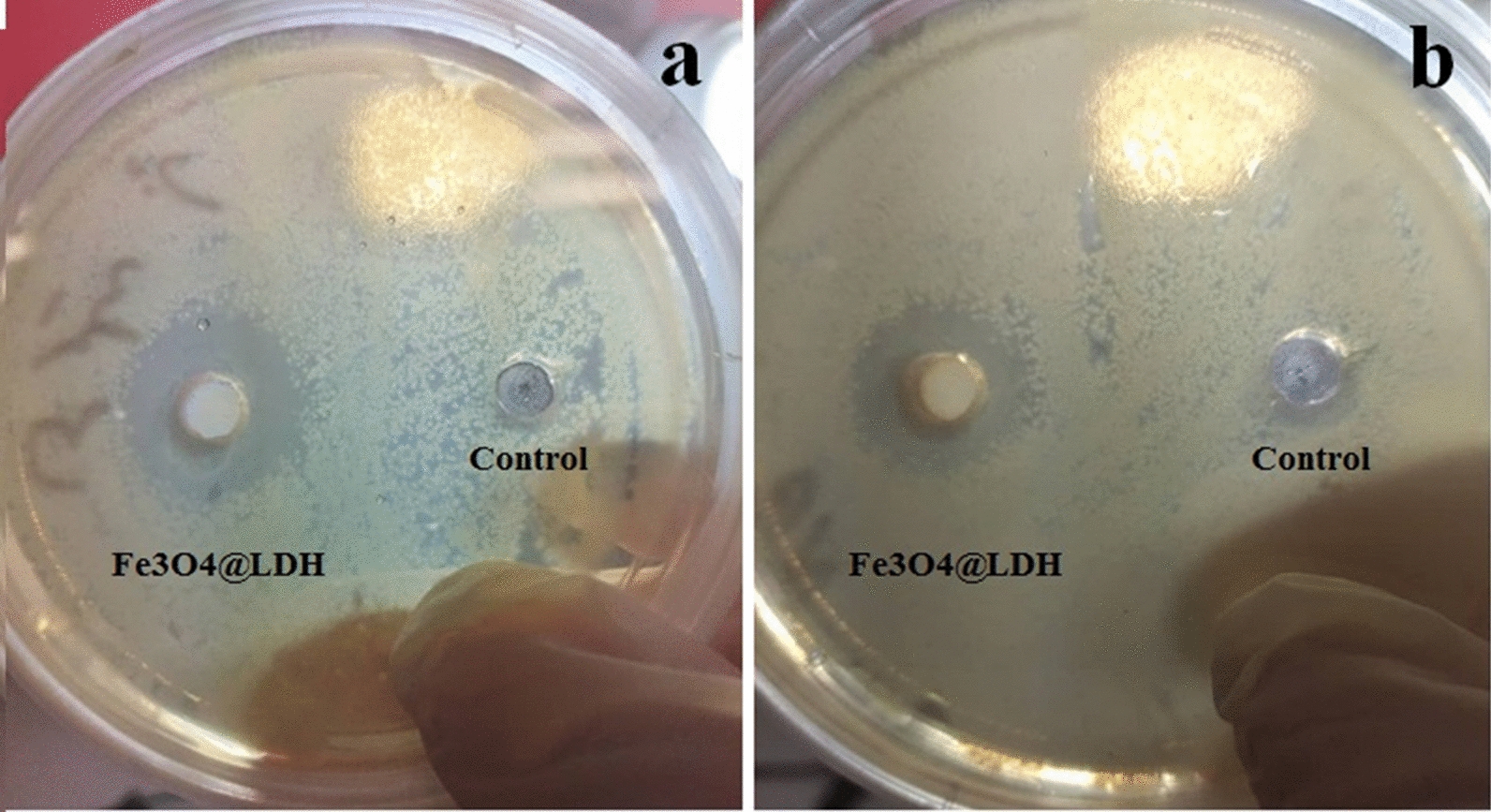
The antagonistic effect of concentrated bacterial cell free culture medium on the growth of **a** *Bacillus cereus* strain ATCC11778^T^ **b** *Klebsiella pneumonia* strain PTCC10031^T^

Inhibition zones for the two pathogen bacteria including *Bacillus cereus* strain ATCC11778^T^ (Fig. 7a) and *Klebsiella pneumonia* strain PTCC10031^T^ (Fig. 7b) were observed in the presence of the nanoparticles. Most nanoparticles and nanostructures exert their antibacterial properties with different mechanisms such as destruction of bacterial membranes, inhibition of biofilm formation, or other multiple mechanisms [24], indicating that the nanoparticle has antimicrobial feature which can be considered as an extra benefit for drug delivery.

**Fig. 8:**
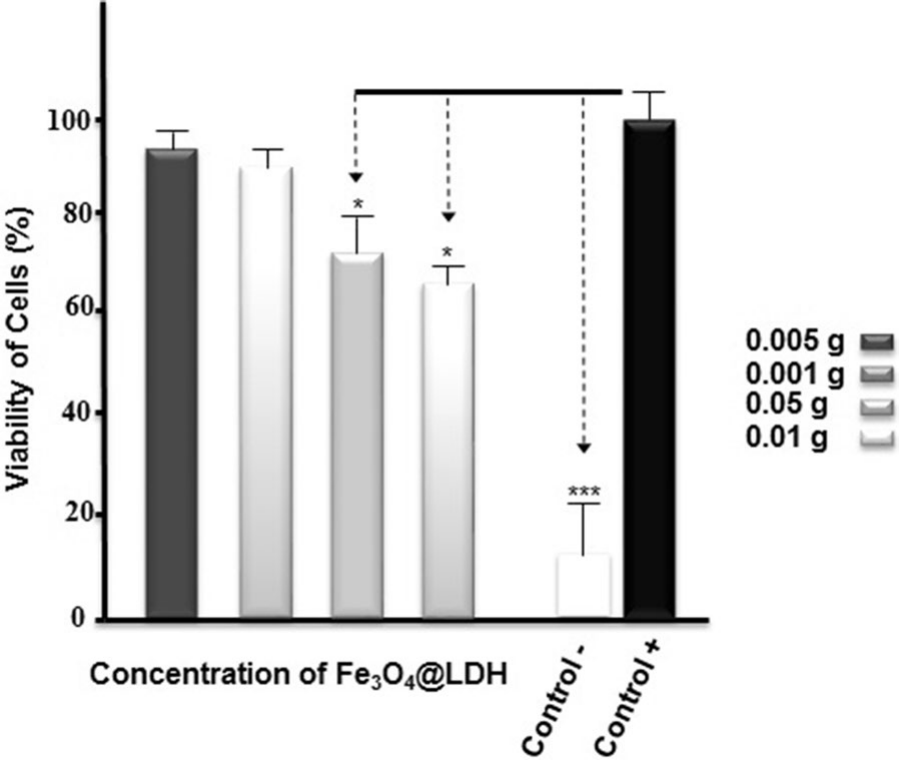
The result of MTT assay in 0.005, 0.001, 0.05 and 0.05 g of Fe_3_O_4_@LDH nanostructure. Data are expressed as mean ± S.E.M. *P < 0.01, **P < 0.005 and ***P < 0.001

MTT assay analysis showed that Fe_3_O_4_@LDH multicore-shell nanostructure in 0.001 g concentration had a less negative effect on C2C12 cells as upon 90% of the cells treated viable in comparison to the control group (Fig. 8). The non-toxicity of nanoparticle can be considered a positive point for using them in drug delivery to eukaryotic organisms, especially humans. In addition, no valuable difference has been observed between 0.001 and 0.005 g concentrations of Fe_3_O_4_@LDH on cell viability. However, it was observed that the viability of about 20–30% of cells decreases in the presence of the high concentration of the nanostructure (0.05 and 0.01 g).

Based on the literature, the nanoparticle size plays an important role in cellular uptake and intracellular trafficking of drug encapsulated in LDH nanoparticles. Several studies have demonstrated that FITC-LDHs are internalized into cells through the clathrin-mediated endocytosis [25]. However, it has been noticed that the mechanism of selectively permeating into cell is effective only at LDH nanoparticle size of 300 nm or less [26]. As the size of Fe_3_O_4_@LDH nanoparticles is 80–130 nm, probably these nanoparticles penetrate into C2C12 cell via selectively clathrin-mediated endocytosis.

### UV–Vis spectrum of Ibuprofen and Diclofenac release assay

Drug release is specified as the speed of mass transport from a solid phase into the broth media under normal conditions. The major phase in drug delivery is an interaction between the drug carrier and PBS (pH = 7.4) that happens at the interface of carrier and buffer solution, and absorption was measured via UV–Vis Spectrophotometer in specified intervals. According to the previous studies, the suitable pH for releasing drug in oral nanoparticles is in physiological buffers condition (pH 7.4) [27]. Each segment of the gastrointestinal (GI) tract maintains its own characteristic pH level from the acidic stomach lumen (pH 1–3) for digestion to the alkaline duodenum and ileum (pH 6.6–7.5) for the neutralization of chyme. Oral nanoparticles retain a hydrophobic and collapsed state in the stomach due to the protonation of hydroxyl groups and increase the zeta potential. After gastric passage, an increase in pH leads to activation of nanoparticles due to decrease of zeta potential and hydrogen bond breakage of interlayers of LDH [27–30]. It should be noted that Ibuprofen and Diclofenac are weak acids that are not be soluble in acidic media, but should be soluble at pH higher than 6.8 [31–33]. Moreover, at a pH above 9, the increase in the concentration of competing OH^−^ anions is responsible for the observed decrease in the recovery [33]; over ~ 90% of the drugs are released at pH 7.4 in the first several hours. Designed nanoparticles for oral drug delivery such as our nanoparticle undergo a surface charge reversal and decrease zeta potential after gastric passage, hoping that drug release will possibly occur in the alkaline intestinal tract instead. Using our inorganic materials with different densities of positively-charged facilitated loading and trapping of anionic drugs such as Ibuprofen (an anti-inflammatory prodrug for bowel disease) in acidic environments (pH < 3). When the drug-loaded nanoparticles were placed in physiological buffers (pH 7.4), a partial negative surface charge on the nanoparticle was generated; this electrostatic repulsion triggered the sustained release of loaded drugs. According to the previous studies, the release assays were, hence, carried out in physiological buffers condition (pH 7.4) [10, 18] at 37 °C which is similar to the normal body temperature.

**Fig. 9:**
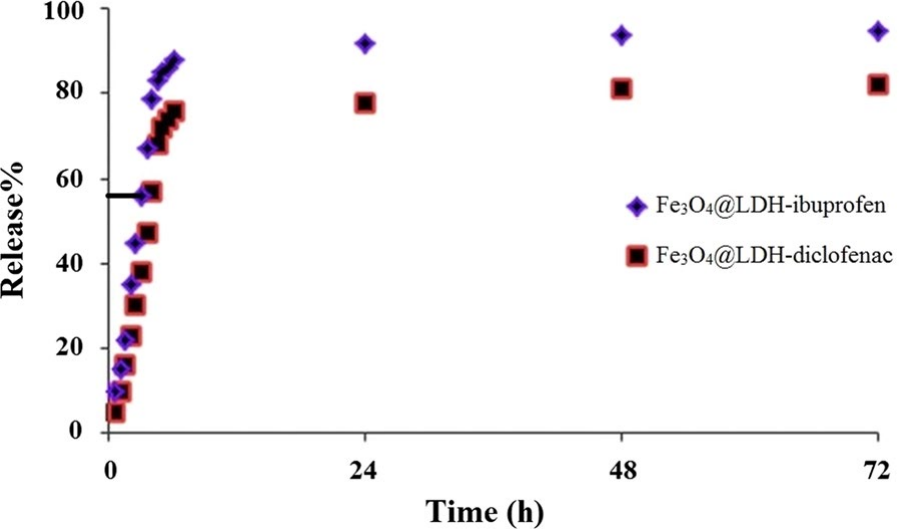
The drug release of Fe_3_O_4_@LDH-ibuprofen and Fe_3_O_4_@LDH-diclofenac at pH = 7.4 and 37 °C

Figure 9 illustrates that the drug release occurred at intervals within 15 min to 72 h in the wavelength of 264 nm for Ibuprofen and wavelength of 276 nm for Diclofenac. Both drug releases gently increased within 15 min to 6 h interval, and the concentration of drug was fixed within 6 h to 72 h interval. The release rate of Ibuprofen was 90% within 24 h, 94% in 48 h, and 96% in 72 h. The values for Diclofenac in 24 h 78%, within 48 h of 81% in 72 h and 82%, respectively, indicating less Diclofenac release in comparison to Ibuprofen per unit of time. This can be due to lower solubility in water [34], highly lipophilicity [35] of Diclofenac, small size, and more sterile effect of Diclofenac compared to Ibuprofen [36] that cannot be easily released between layers. The release gradually arrived the maximum amount of 90% for Ibuprofen and 78% for Diclofenac in the first 6 h. On the other hand, the most absorbed drug in the outer layer of the Fe_3_O_4_@LDH and bonded drug to the substrate by its hydrogen bonds release in the 24 h especially in first 6 h which is useful for quickly developing as a therapeutic dose. Other remaining drugs in the structure less than 10% for Ibuprofen and 20% for Diclofenac which were in the interlayers of the LDH release slowly. The slower delivery rate can be utilized as a therapeutic dose in longer time for decreasing the number of doses required. The cumulative release kinetic of Ibuprofen and Diclofenac from Fe_3_O_4_@LDH nanostructure in phosphate buffer saline (PBS) at pH = 7.4 showed a sustained release of up to 72 h that closely resembled first order release kinetics through a combination of drug diffusion and dissolution of LDH under physiological conditions (Additional file 1: Fig. S3).

Drug loading between LDH layers leads to different release rates of drug and enhances the solubility of the drug and also, reduces its side effects, compared with old and industrial methods.

As other anionic drugs, it seems that the drug release mechanism of Diclofenac and Ibuprofen from the LDH nanoparticles is probably surface diffusion and bulk diffusion via anionic exchange of the drugs anions on, or in, the LDHs with anions in the PBS solution [37]. Fe_3_O_4_/LDHs nanocomposites have been also noticed for drug delivery in different studies due to their layered structure and unique properties [38]. Komarala et al. developed LDH–Fe_3_O_4_ magnetic nanohybrids by a mixed method (coprecipitation synthesis and hydrothermal method) within the range of 10–15 nm for magnetic hyperthermia and delivery of Doxorubicin to cancer cells (HeLa cells) [39]. They showed that Doxorubicin was successfully loaded into the nanohybrids (drug-loading efficiency; ~ 99%) and released by pH dependent manner. The concentration of 0.94 mg/ml (R2 = 0.957) Dox-loaded nanocomposites decreased the viable cell population by 50% and prevented their proliferation [40]. In another study, Fe_3_O_4_@MTX-LDH/Au nanoparticles were developed by Zhao et al. through coprecipitation electrostatic interaction strategy to deliver the anticancer drug of methotrexate (MTX). Likewise, the cumulative percent of the prepared sample and some previously reported materials for Diclofenac and Ibuprofen are compared in Table 1.

**Table 1:** Comparison of different nanocarriers for Diclofenac sodium and Ibuprofen

Nanocarrier	Drug	Max. release (%)	Max. time	pH	Particle size	Refs.
Zn-Al layered double hydroxide (LDH)	Diclofenac sodium	80	60 h	7.4	632.6 nm	[41]
Sodium alginate/layered double hydroxides	Diclofenac sodium	99	8 h	7.4	ND	[42]
Ca–Al layered double hydroxide	Diclofenac sodium	84	400 min	4.6	1–3 µm	[43]
Zn–Al–NO_3_-layered double hydroxides	Diclofenac sodium	98.4	6 h	6.8	132 ± 9 nm	[44]
IBU–LZH	Ibuprofen	77	1200 min	4.8	50–100 nm	[45]
Drug-LDH loaded PCL fibers	Ibuprofen	45	120 h	7.4	147 ± 37 nm	[46]
Alginate–zein/layered double hydroxide biocomposite	Ibuprofen	97	8 h	7.4	ND	[47]
Magnetic@layered double hydroxide	Diclofenac sodium	82	72 h	7.4	80–130 nm	This study
	Ibuprofen	96		7.4		

*ND* not detected

## Conclusion

In this research, Fe_3_O_4_@LDH multicore-shell nanostructure was synthesized through the coprecipitation experiment and used as a new drug nanocarrier. Antimicrobial activity of three important pathogenic bacteria were tested. Fe_3_O_4_@LDH can inhibit the growth of two strains of examined bacteria. This antibacterial property may be due to disruption of bacterial membranes and the hindrance of biofilm formation. Furthermore, nanostructure cytotoxicity was examined on C2C12 myoblast cells via MTT assay analysis. The results revealed that nanostructure in physiological concentration has not cytotoxicity of eukaryotic cells in vitro and can be a good candidate as a nanocarrier for drug delivery in human bodies. Ibuprofen and Diclofenac were selected as the model drugs, being intercalated between LDH nanoflakes to synthesize Fe_3_O_4_@LDH-ibuprofen and Fe_3_O_4_@LDH-diclofenac. Characterization of the synthesized-nanocarrier was achieved using XRD, FT-IR, SEM, VSM, and TEM for displaying the chemical structure and morphology. The Fe_3_O_4_@LDH-ibuprofen and Fe_3_O_4_@LDH-diclofenac were tested for the controlled release of Ibuprofen and Diclofenac under physiological conditions, pH 7.4 (PBS). The above nanostructures also had the advantage of a strong interaction between the carboxyl groups with higher trivalent cations via bridging bidentate, clarity, and high thermal stability. Fe_3_O_4_@LDH multicore-shell nanostructure will possibly have a potential application for constant drug delivery.

## Supplementary information

**Additional file 1: Figure S1.** The standard curve of Ibuprofen in phosphate buffer, pH 7.4. **Figure S2.** The standard curve of Diclofenac sodium in phosphate buffer, pH 7.4. **Figure S3.** The cumulative release kinetic of Ibuprofen and Diclofenac from Fe_3_O_4_@LDH ‎nanostructure in phosphate buffer saline (PBS) at pH = 7.4.

## Data Availability

All data used to generate these results is available in the main text.
