# Supplementary material for: Synthesis and application of magnetic@layered double hydroxide as an anti-inflammatory drugs nanocarrier
Source: J Nanobiotechnology. 2020 Oct 29;18:155. doi: 10.1186/s12951-020-00718-y (PMC7596963; doi:10.1186/s12951-020-00718-y)

**Synthesis and Application of Magnetic@layered Double Hydroxide as an Anti-Inflammatory Drugs Nanocarrier**

Vahid Yousefi^1Ϯ^, Vahideh Tarhriz^1Ϯ^, Shirin Eyvazi^2^ and Azita Dilmaghani^3,4*^

*^1^Molecular Medicine Research Center, Biomedicine Institute, Tabriz University of Medical Sciences, Tabriz, Iran*

*^2^Department of Biotechnology, School of Advanced Technologies in Medicine, Shahid Beheshti University of Medical Sciences, Tehran, Iran.*

*^3^Drug Applied Research Center, Tabriz University of Medical Sciences, Tabriz, Iran.‎*

*‎^4^Faculty of Pharmacy, Tabriz University of Medical Sciences, Tabriz, Iran.‎*

^*^ Corresponding author:

**Azita Dilmaghani, PhD**

Department of Pharmaceutical Biotechnology

Faculty of Pharmacy

Tabriz University of Medical Sciences

Tabriz, Iran

**Email:** [dilmaghani.a@gmail.com](mailto:dilmaghani.a@gmail.com)

^Ϯ^ Vahid Yousefi and Vahideh Tarhriz contributed equally to this work and should be considered as co-first authors.

**Fig. S1.** The standard curve of ibuprofen in phosphate buffer, pH 7.4

**Fig. S2.** The standard curve of diclofenac sodium in phosphate buffer, pH 7.4

Fig. S3. The cumulative release kinetic of Ibuprofen and Diclofenac from Fe3O4@LDH ‎nanostructure in phosphate buffer saline (PBS) at pH=7.4


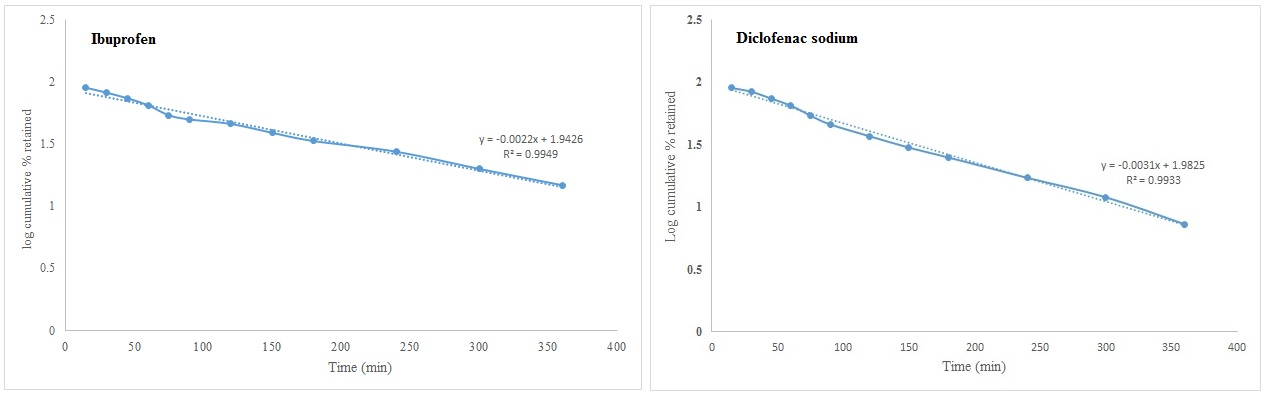

Supplement: Supplementary file 1 — Additional file 1: Figure S1. The standard curve of Ibuprofen in phosphate buffer, pH 7.4. Figure S2. The standard curve of Diclofenac sodium in phosphate buffer, pH 7.4. Figure S3. The cumulative release kinetic of Ibuprofen and Diclofenac from Fe3O4@LDH ‎nanostructure in phosphate buffer saline (PBS) at pH = 7.4. [file 12951_2020_718_MOESM1_ESM.docx]
